# FGF10 Signaling in Heart Development, Homeostasis, Disease and Repair

**DOI:** 10.3389/fgene.2018.00599

**Published:** 2018-11-28

**Authors:** Fabien Hubert, Sandy M. Payan, Francesca Rochais

**Affiliations:** Aix-Marseille Univ, INSERM, MMG, U1251, Marseille, France

**Keywords:** FGF10, FGFR1/2, heart development, cardiomyocyte, cardiac regeneration

## Abstract

Essential muscular organ that provides the whole body with oxygen and nutrients, the heart is the first organ to function during embryonic development. Cardiovascular diseases, including acquired and congenital heart defects, are the leading cause of mortality in industrialized countries. Fibroblast Growth Factors (FGFs) are involved in a variety of cellular responses including proliferation, differentiation, and migration. Among the 22 human/mouse FGFs, the secreted FGF10 ligand through the binding of its specific receptors (FGFR1b and FGFR2b) and subsequent activation of downstream signaling is known to play essential role in cardiac development, homeostasis and disease. FGF10 is one of the major marker of the early cardiac progenitor cells and a crucial regulator of differentiated cardiomyocyte proliferation in the developing embryo. Increasing evidence support the hypothesis that a detailed understanding of developmental processes is essential to identify targets for cardiac repair and regeneration. Indeed the activation of resident cardiomyocyte proliferation together with the injection of cardiac progenitors represent the most promising therapeutical strategies for cardiac regenerative medicine. The recent findings showing that FGF10 promotes adult cardiomyocyte cell cycle reentry and directs stem cell differentiation and cell reprogramming toward the cardiogenic lineage provide new insights into therapeutical strategies for cardiac regeneration and repair.

## Introduction

The heart is an essential muscular organ that pumps blood and provides the whole body with oxygen and nutrients. During embryonic development, the heart is the first organ to form and cardiac morphogenesis is a tightly regulated process. Cardiovascular diseases including congenital and acquired heart diseases are the leading cause of mortality in industrialized countries (Writing Group Members et al., 2016).

By mediating a variety of cellular responses, Fibroblast Growth Factors (FGFs) are known to play an essential role in cardiac development, homeostasis and disease. The human/mouse FGF family comprises 22 members including secreted and intracellular FGFs. Secreted FGFs bind and activate cell surface tyrosine kinase receptors (FGF receptors; FGFRs) encoded by four genes (FGFR1-4). The alternate splicing of FGFR genes results in the generation of seven different receptors, each of them displaying distinct ligand-binding properties (Zhang et al., 2006). In contrast to secreted FGFs, intracellular FGFs serve as cofactors for voltage gated sodium channels and other molecules (Ornitz and Itoh, 2015). Interaction between secreted FGFs and their specific receptors is tightly regulated by extracellular binding proteins including heparan sulfates and the Klotho family proteins that serve as cofactors and confer unique ligand-receptor binding properties. Activated tyrosine kinase FGF receptors mediate diverse intracellular signaling cascades including the RAS-MAPK, PI3K-AKT, PLCγ, and STAT signaling pathways (Ornitz and Itoh, 2015). Phylogenetic analysis suggest that secreted FGFs can be grouped into five subfamilies of paracrine FGFs and one subfamily of endocrine FGFs. The current consensus suggests that FGF10 belongs to a subfamily that comprises FGF3, FGF7, FGF10, and FGF22. Receptor-ligand specificities are well described. Indeed, FGF3, 7, 10, and 22 have been shown to activate preferentially the IIIb splice variant of FGFR2. In addition FGF3 and FGF10 also activate the IIIb splice variant of FGFR1 (Zhang et al., 2006). Nevertheless, ablation studies together with overlapping expression patterns strongly suggest potential functional redundancy between FGF family members in the developing and adult heart. Finally, the existence of heterodimer formation between FGFs and FGFRs may further increase receptor-ligand interaction possibilities (Sun et al., 2002) and thus the diversity of FGF signaling.

Here we will review a detailed understanding of FGF signaling in cardiovascular development, homeostasis, disease and repair, focusing on the particular role of the FGF10/FGFR1/FGFR2 pathway.

## Developmental Role of the Fgf10 Signaling

Heart development is an extremely complex process that can be divided in two major growth phases distinguished by a shift in the major site of proliferation from an extracardiac progenitor cell population to fetal cardiomyocytes. The early embryonic phase relies on the extensive proliferation of cardiac progenitor cells termed the second heart field (SHF) and their progressive addition to the developing heart tube. Precise spatiotemporal control of SHF progenitor cell proliferation-differentiation balance is required for normal heart tube elongation. Cardiac neural crest (CNC) cells, a second extracardiac cell population, play a critical role in early heart development (Hutson and Kirby, 2007). Concomitant with SHF cell addition to the outflow tract (OFT) of the heart, CNC migrate from the dorsal neural tube into the OFT. Interactions between CNC cells and SHF progenitors are critical determinants for the correct addition of SHF cells to the heart tube. In contrast to early heart tube development, fetal heart growth is achieved through the proliferation of differentiated cardiomyocytes which tight control is essential for the correct morphogenesis of the heart. Indeed, perturbations in the regulation of fetal cardiomyocyte proliferation lead to congenital heart defects.

During the early embryonic phase of heart morphogenesis, proper communication between cardiac progenitor cells is a prerequisite for correct heart tube elongation, looping, and arterial pole alignment. FGFs are among the critical signals required for proper early cardiac morphogenesis (Kelly, 2012). By ensuring communication within and between developing heart progenitors, FGF signaling leads to their tight regulation of proliferation and specification. Indeed, transgenic mouse models with conditional inactivation of *Fgfr1/2*, conditional overexpression of *Sprouty2* (*Spry2*, which encodes an FGF signaling antagonist) or conditional ablation of *Frs2* (encoding a MAPK/PI3K signaling adaptor protein) within the SHF progenitor cell population revealed that interrupting autocrine FGF signaling in SHF mesoderm, by compromising SHF progenitor cell proliferation and by indirectly reducing cardiac neural crest cell recruitment into the outflow tract cushions, causes outflow tract misalignment and subsequently impaired cardiac morphogenesis (Park et al., 2008; Zhang et al., 2008). While FGFR-dependent regulation of SHF proliferation seems to depend on the PI3K/AKT pathway (Luo et al., 2015), the Ras/Erk downstream signaling seems to be required in the regulation of myocardial specification (Rochais et al., 2009; Hutson et al., 2010). All these studies thus strongly reveal iterative roles for FGF signaling in OFT development.

**FIGURE 1:**
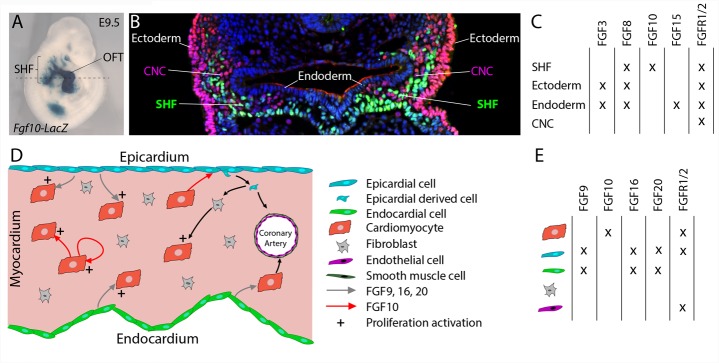
FGF10 signaling in the developing heart. **(A)** Lateral whole-mount view and **(B)** transverse section of an embryo carrying an *Fgf10-LacZ* transgene (Kelly et al., 2001) at embryonic day E9.5. *Fgf10* transgene expression is observed in second heart field (SHF) progenitor cells, which are located in pharyngeal mesoderm adjacent to pharyngeal endoderm, and in the outflow tract (OFT). **(B)** Immunofluorescence on transverse section of an E9.5 embryo carrying an *Fgf10-LacZ* transgene, at the level of the dotted line in **(A)**. The anti-AP-2α (pink) antibody was used to detect cardiac neural crest (CNC) cells and ectodermal cells and the anti-β galactosidase (green) antibody to visualize SHF cells. **(C)** Table showing the overlapping expression patterns of key FGF ligands and receptors at E9.5 in the SHF and surrounding tissues. **(D)** FGF signaling role in fetal heart development. **(E)** Table showing the overlapping expression patterns of key FGF ligands and receptors in the fetal heart.

Multiple FGF ligands have been described to be expressed in cardiac progenitors and surrounding tissues (Figures 1A–C). FGF10 was identified as a specific endogenous marker of the SHF (Kelly et al., 2001). While *Fgf10* expression is restricted to SHF progenitors (Kelly et al., 2001), *Fgf8* is also expressed in the adjacent pharyngeal ectoderm and endoderm (Ilagan et al., 2006; Mesbah et al., 2012). *Fgf15* expression has been detected in the pharyngeal endoderm (Vincentz et al., 2005) and *Fgf3* is expressed in the pharyngeal endoderm and ectoderm (Urness et al., 2011).

The Wnt/β-catenin signaling pathway a key regulator of SHF development transcriptionally controls *Fgf10* expression within SHF progenitors (Cohen et al., 2007). Crucial transcription factors of SHF cardiac progenitor cell deployment are also known to control *Fgf10* expression. ISL1 and NKX2-5 control the expression of *Fgf10* in the SHF, through competitive binding to common regulatory elements in an intronic cardiac enhancer, thus, respectively, activating expression in progenitor cells and repressing transcription in differentiated myocytes (Watanabe et al., 2012). TBX1 also activates *Fgf10* through T-box binding sites in the same enhancer element (Watanabe et al., 2012).

*Fgf10*-null embryos, which die at birth due to lung aplasia, display altered heart morphology. In addition to the absence of pulmonary arteries and veins, *Fgf10* knockout embryos display an abnormal positioning of the ventricular apex in the thoracic cavity (Marguerie et al., 2006; Rochais et al., 2014). Nevertheless, early SHF deployment and subsequent heart tube elongation are not affected by *Fgf10* deletion. In contrast, deletion of the main FGF10 receptor, *Fgfr2b*, leads to major congenital heart defects including ventricular septal defects, OFT alignment defects, and thin and poorly trabeculated ventricles (Marguerie et al., 2006) strongly suggesting the existence of functional redundancy between FGF10 and other FGFR2b ligands during the early steps of heart development. FGF8 appears to be the major ligand regulating cardiac progenitor cell deployment. A series of conditional loss of function experiments has revealed that *Fgf8*, through a cell-autonomous mechanism, is required for SHF expansion and thus OFT elongation, septation, and subsequent ventriculoarterial alignment (Ilagan et al., 2006; Park et al., 2008). Interestingly, the fact that heterozygous deletion of *Fgf10* in combination with homozygous loss of mesodermal *Fgf8* expression results in more severely altered anterior heart development (Watanabe et al., 2010) strongly supports mesodermal FGF8 and FGF10 functional redundancy. In addition, FGF3 and FGF10 have been also shown to play redundant and dosage sensitive requirement during heart tube elongation (Urness et al., 2011). All these studies highlight that critical FGF dosage, including FGF10, is crucial for SHF proliferation and deployment and thus for normal cardiac morphogenesis.

During the second phase of heart development (after embryonic day E10.5), subsequent growth and remodeling of the myocardium occur without significant further addition of cardiac progenitor cells to the heart. Instead, regulated proliferation of cardiac myocytes drives growth of the atrial and ventricular chambers. Tight spatio-temporal regulation of fetal cardiomyocyte proliferation thus appears to be required for proper heart formation and impairment of cardiomyocyte proliferation during fetal stages also results in congenital heart defects (Ahuja et al., 2007). FGF signals, through cell-autonomous or paracrine mechanisms, have been described as crucial regulators of fetal cardiomyocyte proliferation (Figures 1D,E; Smith and Bader, 2007). FGF ligands originating from the endocardium and the epicardium, including FGF9, FGF16, and FGF20, have been shown to regulate cardiomyocyte proliferation (Lavine et al., 2005; Hotta et al., 2008; Lu et al., 2008). Recent reports revealed the implication of FGF10 in the regulation of fetal cardiomyocyte proliferation. *Fgf10* mutant heart analysis demonstrates that FGF10 signaling, through a cell-type autonomous mechanism, specifically controls fetal right ventricular cardiomyocyte proliferation. In fact, at fetal stages, FGF10/FGFR2b signaling promotes cardiomyocyte proliferation through the phosphorylation of the FOXO3 transcription factor and subsequent downregulation of the cyclin dependent kinase inhibitor p27^kip1^ expression (Rochais et al., 2014). In addition, myocardial FGF10 signaling, through the paracrine activation of FGFR1 and FGFR2 in the epicardium, has been suggested to promote epicardial-derived cell migration into the compact myocardial layer (Vega-Hernandez et al., 2011). In this study, the impairment in cardiac fibroblast numbers observed in *Fgf10*-mutant hearts, results indirectly in reduced fetal cardiomyocyte proliferation.

Despite cardiomyocyte proliferation, FGF signals, through redundant function of FGFR1 and FGFR2, originating from the epicardium and the endocardium, play pivotal role in coronary vasculature development (Figure 1D). In fact, in embryonic mouse heart, myocardial FGFR1/2 signaling by triggering Hedgehog signaling activation, *Vegf* and *Angiopoietin-2* expression, indirectly participate to the coronary vascular plexus formation and thus coronary vessel deployment (Lavine et al., 2006). Here the precise requirement of the FGF10 ligand has not been explored.

Several members of the FGF family are expressed in the vascular network (Presta et al., 2005; Beenken and Mohammadi, 2009). While the most studied FGF member, FGF2, is a potent inducer of angiogenesis, other FGFs (FGF 1, 2, 5, 7, 8, 16, and 18), but not FGF10, are expressed in endothelial and vascular smooth muscle cells (Antoine et al., 2005). Despite predominant FGFR1 and FGFR2 expression endothelial cells (Presta et al., 2005), mouse specific deletion of *Fgfr1* and *Fgfr2* in both endothelial and hematopoietic cell lineages has no impact on normal vascular development (Oladipupo et al., 2014; House et al., 2016). In contrast, in zebrafish, global FGFR signaling inhibition using allosteric inhibitor or a dominant negative form of the receptor revealed the critical requirement for FGF signaling in the maintenance vascular function and integrity (Murakami et al., 2008; De Smet et al., 2014). This strongly suggests, in mammals, the existence of functional redundancy between FGFR1, FGFR2, and FGFR3 that also expressed in endothelial cells.

## Fgf10 Signaling in Cardiac Homeostasis

Soon after birth, the ability of cardiomyocytes to proliferate is strongly reduced, and cardiac growth transitions from hyperplastic to hypertrophic (Pasumarthi and Field, 2002). For nearly a century, the adult heart has been considered to be a post-mitotic organ; however, recent studies have highlighted the importance of the homeostasis of the adult heart in physiological conditions. Indeed extensive studies on adult mammalian hearts including the human heart have led to a consensus that new cardiomyocytes are indeed generated throughout life (Soonpaa and Field, 1998; Bergmann et al., 2009, 2015). In the healthy adult murine and human heart, cardiomyocyte renewal is currently estimated at 0.5–2% per year (Eschenhagen et al., 2017).

**FIGURE 2:**
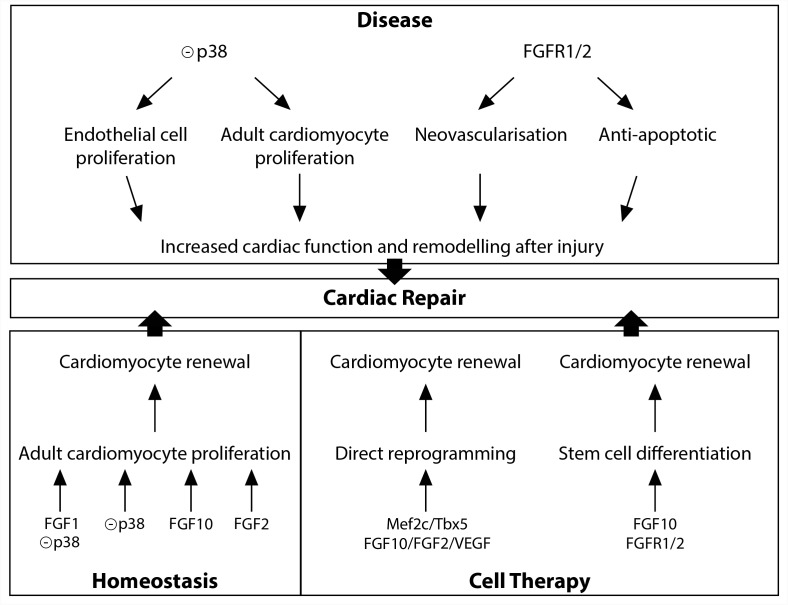
FGF10 signaling in cardiac homeostasis, disease and repair.

Diverse FGFs and downstream signals, including FGF1, FGF2, FGF10, and p38 MAP kinase have been shown to be involved in the regulation of adult cardiomyocyte renewal (Figure 2). *In vitro* studies initially described FGF2 as a potent positive regulator of cardiomyocyte proliferation (Pasumarthi and Field, 2002). *In vitro* and *in vivo* experiments indicate that p38 MAP kinase inhibition alone (Jopling et al., 2012b) or in combination with FGF1 treatment (Engel et al., 2005, 2006), leads to partial cardiomyocyte dedifferentiation and cell-cycle progression. Furthermore, in the adult zebrafish, epicardial cells addition to the ventricle has been shown to support cardiac homeostasis in an FGF-dependent fashion (Wills et al., 2008). Finally, in the adult mouse heart, FGF10 has been described to be a potent regulator of cardiomyocyte proliferation. Indeed, temporal *Fgf10* overexpression rapidly enhanced adult cardiomyocyte cell cycle re-entry leading to increased ventricular wall thickness. While FGF10 regulation of fetal cardiomyocyte proliferation seems to occur through the FGFR2b, FGF10 may activate predominantly the FGFR1b to promote adult cardiomyocyte proliferation (Rochais et al., 2014).

## Implication of Fgf10 Signaling in Cardiovascular Diseases and Repair

Cardiovascular diseases are the leading cause of mortality in industrialized countries (Writing Group Members et al., 2016). Characterized by any molecular, cellular and physiological change in the myocardium, coronary vessels or valves, cardiac diseases result in cardiomyocyte loss and impaired cardiac function that ultimately lead to congestive heart failure. Multiple FGFs including FGF10 signaling have been described to play pathophysiological roles in the cardiovascular system (Itoh et al., 2016).

Diverse studies highlighted a role for the FGFR1/2 signaling in the neovascularization after injury (Figure 2). In the zebrafish injured heart, epicardial *Fgfr2* expression is upregulated and FGFR signaling blockade leads to a failure in coronary neovascularization, resulting in severely impaired cardiac regeneration (Lepilina et al., 2006). In addition, neovascularization and vascular remodeling in response to injury is severely impaired in endothelial specific FGFR1/2 deficient mice (Oladipupo et al., 2014; House et al., 2016). Finally, endothelium-targeted overexpression of constitutively active FGFR2 post-myocardial infarction results in anti-apoptotic action with enhanced angiogenesis (Matsunaga et al., 2009).

While zebrafish adult heart fully regenerates after injury (Poss et al., 2002), damaged adult mammalian myocardium is replaced by fibrotic scar tissue. The MAPK pathway plays a crucial role in adult zebrafish heart regeneration (Figure 2). Indeed, the induction of p38 MAPK activity prevents cardiomyocyte proliferation and subsequent heart regeneration (Jopling et al., 2012a). In the adult mouse heart, p38 inhibitor injection, after acute myocardial injury, enhances cardiomyocyte and endothelial cell proliferation and preserves cardiac remodeling and function (Engel et al., 2006) strongly revealing that downstream FGF signaling may be beneficial to improve the limited innate regenerative capacities of the adult mammalian heart. In contrast to the adult heart, neonatal mammalian heart, including mouse, pig and human, possesses extensive regenerative capacities (Porrello et al., 2011; Haubner et al., 2016; Zhu et al., 2018). Nevertheless, the rapid and dramatic decrease in cardiomyocyte proliferation rate during the first week of postnatal life (Pasumarthi and Field, 2002) results in severely limited regenerative capacities in adult, strongly supporting the hypothesis that a detailed understanding of the regulation of fetal cardiomyocyte proliferation is essential to identify targets for cardiac regeneration. As described above, FGF10 has been identified as a crucial regulator of fetal cardiomyocyte proliferation (Rochais et al., 2014). The fact that decreased myocardial *Fgf10* expression has been observed in mouse postnatal heart during the time window where cardiomyocytes exit from the cell cycle (Rochais et al., 2014), coinciding with the loss of regenerative capacities, suggests that FGF10 signaling may play a role in cardiac regeneration. However, *Fgf10* overexpression in the neonatal mouse heart does not promote beneficial effects on post-natal cardiac regeneration (Rubin et al., 2013). Nevertheless, the ability for FGF10 to specifically induce adult cardiomyocyte cell-cycle reentry in physiological conditions suggests that FGF10 might be able to promote cardiomyocyte renewal in the adult injured heart (Rochais et al., 2014).

Together with the stimulation of existing cardiomyocyte renewal, cell therapy using the injection or tissue-based implantation of cardiac progenitor cells and direct reprogramming represent relevant therapeutic options for cardiac regenerative medicine (Tzahor and Poss, 2017). Several studies revealed the requirement of FGF10 signaling during stem cell specification into the cardiogenic lineage (Figure 2). Indeed, FGF10 signaling has been shown to play an important role in promoting cardiomyocyte differentiation in both embryonic and induced pluripotent stem cells (Chan et al., 2010). Furthermore, in addition to improve the quality of cardiac reprogramming in mouse fibroblasts, and in combination with FGF2 and the vascular endothelial growth factor (VEGF), FGF10, through the RAS-MAPK and PI3K/AKT pathways, is able to convert partially reprogrammed cells into functional cardiomyocyte-like cells (Yamakawa et al., 2015).

## Conclusion

All the studies described in this review highlighted the crucial role for the FGF10 ligand and the related FGFR1/2 signaling in heart development, homeostasis and disease. The recent findings revealing a crucial role for FGF10 in controlling both adult cardiomyocyte cell cycle reentry and stem cell differentiation and cell reprogramming toward the cardiogenic lineage provide potential therapeutic strategies for cardiovascular diseases.

## Author Contributions

FH, SP, and FR wrote the manuscript.

## Conflict of Interest Statement

The authors declare that the research was conducted in the absence of any commercial or financial relationships that could be construed as a potential conflict of interest.
